# Eccentric training at long muscle lengths induces greater corticospinal and spinal reflex plasticity than eccentric training at short muscle lengths

**DOI:** 10.1113/EP092470

**Published:** 2025-06-02

**Authors:** Julian Colard, Yohan Betus, Tristan Tallio, Baptiste Bizet, Antoine Nordez, Marc Jubeau, Thomas Cattagni

**Affiliations:** ^1^ Nantes Université, Movement–Interactions–Performance (MIP), UR 4334 Nantes France

**Keywords:** corticospinal, H reflex, lengthening, neural plasticity, SICI, spinal

## Abstract

It is well‐established that resistance training generates neural adaptations. These may be greater when exercises mainly include eccentric contractions or when muscles are trained at long length. However, it remains to be clarified whether the length at which muscles are trained influences neural adaptation following eccentric training. We trained 28 healthy young individuals with eccentric exercises for 9 weeks (24 sessions) at either short (*n* = 13) or long (*n* = 15) plantar flexor lengths. Participants were assessed once before and once after this training. Estimates of corticospinal excitability and short‐interval intracortical inhibition were obtained using transcranial magnetic stimulation and by analysing conditioned or non‐conditioned motor evoked potentials. Effectiveness of Ia afferents to discharge α‐motoneurons, and post‐activation depression induced by primary afferent depolarization were estimated using peripheral tibial nerve stimulation conditioned or not by fibular nerve stimulation, and by analysing Hoffmann reflex amplitude. Maximal plantar flexor torque and voluntary activation were also assessed. The increase in corticospinal excitability and effectiveness of Ia afferents to discharge α‐motoneurons were significantly greater after training at long muscle length than at short muscle length (+24.03% and +16.1%, respectively, *P* < 0.001), without between‐group differences in adaptations for short‐interval intracortical inhibition, post‐activation depression by primary afferent depolarization, maximal torque or voluntary activation level. These results suggest that eccentric training performed at long muscle lengths induces greater adaptations in corticospinal and spinal reflex plasticity. It is crucial to consider muscle length during eccentric training to optimize neuronal plasticity and potentially enhance daily task performances.

## Abstract (highlights)

- **What is the central question of this study?**
  Does the use of different muscle lengths (short and long) influence the neural adaptations observed following eccentric training of the plantar flexors?
- **What is the main finding and its importance?**
  Eccentric training at long muscle lengths induces greater corticospinal excitability and spinal reflex adaptations than eccentric training at short muscle lengths. This supports the notion that muscle length is a crucial factor in promoting neural plasticity, which is an important consideration in the design of eccentric training programmes.

## 1 INTRODUCTION

Optimizing strength gains and improving neuromuscular performance are fundamental objectives in the field of sports science and rehabilitation. Among the different types of muscle conditioning, eccentric training has emerged as a powerful method for promoting strength gains (Guilhem et al., 2010) through unique physiological adaptations, from brain to muscle (Duclay et al., 2008). Eccentric training involves contractions performed when a force applied to a muscle exceeds the momentary force produced by the muscle itself, resulting in the forced lengthening of the muscle–tendon system while contracting (Lindstedt et al., 2001). Despite the importance of muscle length in muscle structural adaptations related to eccentric training (Guex et al., 2016; Marušič et al., 2020), its influence on neural adaptations needs to be clarified. This knowledge would be useful to determine whether muscle length offers significant physiological advantages that should be considered in eccentric training.

Part of the physiological adaptations that take place following resistance training are attributed to neural adaptations (Enoka, 1988; Moritani & deVries, 1979). Briefly, resistance training may increase excitability of the main motor pathways, thanks to adaptations in both the brain and the spinal cord (Siddique et al., 2020; Škarabot et al., 2021). These adaptations contribute to enhancing neuromuscular activation through mechanisms such as a decrease in recruitment threshold and an increased firing rate of motor units, ultimately resulting in strength gains. Evidence has shown that resistance training based on eccentric contractions is particularly efficient for improving neural adaptations, voluntary drive and then strength gains (Duclay et al., 2008; Škarabot et al., 2021). Indeed, eccentric training induces greater strength gains than concentric training, particularly when strength is measured during eccentric contractions (Farthing & Chilibeck, 2003; Hedayatpour & Falla, 2015; Pakosz et al., 2023; Roig et al., 2009). Neural adaptations from brain to spinal levels could explain this difference. It is reported that the increase in corticospinal excitability is generally greater after eccentric compared to concentric exercise (Latella et al., 2019), and after eccentric compared to concentric resistance training (Kidgell et al., 2015). However, some studies have yielded less pronounced outcomes (Carroll et al., 2002). This result is associated with a greater reduction of intracortical inhibition after eccentric training than after concentric training (Kidgell et al., 2015). Eccentric training, but not concentric training, enhances the effectiveness of activated Ia afferents to discharge α‐motoneurons in the soleus (SOL) muscle, particularly during eccentric contractions (Duclay et al., 2008). It is interesting to note that this adaptation occurs for all types of contraction in the gastrocnemius medialis (GM) (Duclay et al., 2008). This suggests the presence of differential spinal adaptations depending on the contraction type and muscle group (Duclay et al., 2008). These adaptations may include pre‐ and/or postsynaptic mechanisms acting on α‐motoneurons.

Muscle length during resistance exercise training also influences physiological adaptations and strength gains. In the quadriceps, hamstrings and biceps brachii, resistance training with long muscle length promotes greater strength gains and hypertrophy than resistance training with short muscle length (Maeo et al., 2021; Pedrosa et al., 2023; Sato et al., 2021). Indeed, Guex et al. (2016) showed that increases in fascicle length and strength gains in the hamstrings were greater with eccentric training performed at long muscle length than at short muscle length. However, the effects of muscle length during eccentric training on neural adaptations are limited. Such a combined effect is possible because eccentric exercise and exercise at long muscle length are strong modulators of neural drive and spinal excitability within the central nervous system (Guex et al., 2016; Marušič et al., 2020). Voluntary activation during maximal contraction shows greater levels at longer muscle lengths compared with shorter ones (Doguet, Rivière et al., 2017). This outcome was partially attributed to an increased descending neural drive that induces greater motoneuron output. Interestingly, intracortical inhibition, commonly reduced during eccentric contractions, is observed to increase at long muscle lengths (Doguet, Nosaka et al., 2017). In addition, muscles fascicles and sarcomeres are strongly stretched in the final part of a contraction range during eccentric contraction (Guilhem et al., 2016). It is therefore possible that the greater muscle length induces different peripheral and central nervous stimuli. Indeed, at greater muscle length, muscle spindle activity is amplified, inducing greater type Ia and II afferent discharges (Dimitriou, 2022; Matthews, 1962). Furthermore, there is a possibility of a difference in the qualitative regulation of supraspinal control on presynaptic regulatory mechanisms during eccentric contractions, particularly with regard to greater primary afferent depolarization (PAD) (Colard et al., 2023). To sum up, the neural control of eccentric contraction is different at long than shorter muscle length. The chronic application of eccentric exercises at long or short muscle lengths could induce different neural adaptations at both cortical and spinal levels.

Our study aimed to determine the effect of eccentric training at different muscle lengths (short vs. long) on neural adaptations in plantar flexors. This muscle group was chosen for methodological reasons and for the quality of its nervous responses (particularly the H reflex) during voluntary contractions. We hypothesized that eccentric training at long muscle length induces greater neural adaptations at both cortical and spinal levels, promoting greater strength gains, than eccentric training at short muscle length.

## 2 METHODS

### 2.1 Ethical approval

The study conformed with standards set by the *Declaration of Helsinki* (2013), except for registration in a database and was approved by the Institutional Review Board of Nantes University (CERNI #06012023). All volunteers gave their informed written consent before participation in the study.

### 2.2 Participants

Thirty participants were initially enrolled in the study and were randomly assigned to two distinct training groups: a group training at short muscle length (SHOgroup; *n* = 15) and a group training at long muscle length (LONgroup; *n* = 15). Two participants from the SHOgroup voluntarily withdrew from the training protocol, resulting in their exclusion from the final analysis. Finally, 13 SHOgroup participants (six females, age 19 ± 1 years, height 172 ± 11 cm, body mass 64 ± 11 kg) and 15 LONgroup participants (five females, age 20 ± 2 years, height 174 ± 11 cm, body mass 66 ± 11 kg) completed the protocol. All participants were students in the faculty of sport sciences of Nantes Université. Inclusion criteria were being healthy and aged between 18 and 30 years. Exclusion criteria were as follows: (i) recent (<3 years) musculoskeletal injury or disability in spine or lower limbs; (ii) cardiovascular, neurological or psychiatric disease; and (iii) recent enrolment in any strength training programme. A previous meta‐analysis (Siddique et al., 2020) demonstrated a moderate effect size for increases in muscle strength following chronic resistance training interventions (standardized mean difference (SMD): 0.67, 95% CI: 0.41–0.94) across multiple studies (*n* = 325; *P* < 0.001), with moderate heterogeneity between results (τ^2^ = 0.33; χ^2^ = 73.89; df = 31; *P* < 0.001; *I* ^2^ = 58%). However, since such effects are unlikely to accurately represent the sample size required for this chronic study on the influence of muscle length on corticomotor responses, we recruited participants based on convenience sampling, consistent with typical sample sizes reported in this field.

### 2.3 Study design

The eccentric training consisted of 24 sessions of eccentric exercise over a maximum 9‐week period (three sessions/week) at either short (SHOgroup) or long (LONgroup) muscle lengths. Participants were tested 3–5 days before the first eccentric training session (pre‐training testing session) and 3–5 days after their last eccentric training session (post‐training testing session). The pre‐training and post‐training testing sessions lasted approximately 3 h. All participants were asked not to perform any strenuous exercise for 48 h before the two testing sessions.

### 2.4 Eccentric training

The progressive nature of the 24 training sessions was ensured by monitoring the changes in maximum concentric repetition (1 repetition maximum; 1RM) every two sessions, which were used as the basis for determining the training load. The training programme was closely monitored and supervised by the same experimenters. Each training session started with a 10‐min warm‐up tailored to the upcoming exercise. Each training session consisted of: (i) eccentric heel raise movements performed on a Smith squat machine (10 sessions), or (ii) eccentric heel raise movements performed on an inclined leg press (10 sessions), or (iii) eccentric plantar flexions performed on an isokinetic dynamometer (sessions 6, 12, 18 and 24; Table 1).

**TABLE 1:** Summary of the eccentric training protocols used in the short‐muscle and long‐muscle length groups.

Modality	Apparatus	Number of sessions	Prescription (series × reps)	Tempo (s) Con–Isom–Ecc–Rest	Recovery between series (s)	Loading dose
Isotonic	Heel drop on the Smith machine	5	5 × 10	1‐1‐3‐1	90	100% of concentric 1RM
		5	1RM assessment 4 × 10	1‐1‐3‐1	90	100% of concentric 1RM
	Heel drop on the oblique press	5	5 × 10	1‐1‐3‐1	90	100% of concentric 1RM
		5	1RM assessment 4 × 10	1‐1‐3‐1	90	100% of concentric 1RM
Isokinetic	Dynamometer	4	5 × 10	1‐1‐3‐1	90	100% eccentric

*Note* that the concentric phase was performed bilaterally, whereas the eccentric phase involved alternating between the right and left sides. Abbreviations: 1RM, one repetition concentric maximum; Con, concentric; Ecc, eccentric; Isom, isometric.

#### 2.4.1 Heel raises on Smith machine and inclined leg press

For the eccentric exercises in the standing heel raise sessions, participants stood upright in the Smith machine, keeping their knees and hips straight, with a bar resting on their shoulders behind the neck (De Azevedo et al., 2023).

To safely execute the heel raise on the inclined leg press, participants flexed their hips to a 90° angle. Only for these exercises participants kept their knees slightly bent, thereby preventing hyperextension while performing the movement.

During the concentric and isometric phases, both groups engaged in bilateral plantar flexion and a unilateral eccentric dorsiflexion. More precisely, after the plantar flexion, the participants performed the dorsiflexion on alternate single legs. The participants followed a 1‐1‐3‐1 rhythm synchronized with a metronome: 1 s for the ascent, 1 s for the isometric hold, 3 s for the descent (eccentric phase) and 1 s of rest. We performed unilateral concentric 1RM tests every other session, ensuring the same joint ranges of motion were used as during the training sessions. The eccentric workload was set at 100% of the concentric unilateral 1RM. Participants executed four sets of 10 repetitions on both right and left sides when their 1RM had been determined at the beginning of the session; in the absence of 1RM determination, participants had to complete five sets. Each set was followed by a 90‐s rest period. The training load was adjusted to match the concentric 1RM.

Only the joint angles differed between the groups. The SHOgroup performed the eccentric dorsiflexion from 30° plantar flexion to 0° (neutral position, with the foot on the floor). The LONgroup executed it from 0° (neutral position) to 30° of dorsiflexion.

To ensure that participants actually performed the specified range of motion for their training group, measuring scales (in cm) were positioned on both the Smith squat and inclined leg press machines. These scales were marked at the start and end of the range of motion based on joint angles measured in static condition using a manual goniometer. As participants could not see these scales, investigators provided guidance to ensure they reached the correct starting position.

#### 2.4.2 Isokinetic dynamometer

For the eccentric exercise performed on the isokinetic dynamometer (sessions 6, 12, 18 and 24), two different systems were used: the Biodex (System 3; Biodex Medical Systems, Upton, NY, USA), and Con‐trex (Con‐Trex MJ; CMV AG, Dübendorf, Switzerland). While seated on the dynamometer chair with their knees fully extended and their hips flexed at 90°, participants were instructed to complete four sets of 10 maximal eccentric contractions of the plantar flexors. The range of motion (i.e. 30°) and joint angles were the same as for the other two exercises. The velocity during eccentric contraction was kept constant (between 10 and 15° s^−1^). Each set was followed by a 90‐s rest period.

### 2.5 Measurements

#### 2.5.1 Mechanical data

The participants were seated with their trunk inclined at 30° and knee joints extended at 0° (representing full extension). Measurements were taken on the right foot fixed to the footplate of an isokinetic dynamometer (Biodex system 3; Biodex Medical Systems, Shirley, NY, USA), which enables an accurate recording of muscle torque at a consistent angular velocity. The dynamometer output signal was sampled at 2 kHz via a commercial acquisition system (CED Power 1401‐3A; Cambridge Electronic Design, Cambridge, UK), displayed and stored using Signal 7 software (Cambridge Electronic Design). The dynamometer axis of rotation was meticulously aligned with the anatomical ankle flexion–extension axis. Stability of participants was ensured through the use of a neck brace secured to the seat, chest belts and an abdominal belt. Particular attention was paid to maintaining their posture to minimize external sensory influences. This approach aimed to maintain consistent corticovestibular influences on motor pool excitability while reducing afferent feedback from other peripheral receptors, including Golgi tendon organs, cutaneous receptors and joint afferents (Duclay et al., 2014; Zehr, 2002). During pre‐ and post‐sessions all measurements related to the evaluation of evoked potentials were collected at 0° (ankle angle) during dynamic and isometric contractions. For dynamic conditions, an angle range of motion of 30° from 15° (plantar flexion) to −15° (dorsiflexion) was used. The velocity during each contraction was kept constant (10° s^−1^). Eccentric and isometric contraction types were studied separately in randomized order (further details in the ‘Experimental protocol’ section).

#### 2.5.2 Electromyography

Electromyographic (EMG) signals were recorded on SOL, GM and tibialis anterior (TA) muscles with pairs of self‐adhesive surface electrodes (Meditrace 100; Covidien, Mansfield, MA, USA) in bipolar configuration with a 30 mm inter‐electrode distance. SOL electrodes were placed 2 cm below the muscle–tendon junction of the gastrocnemii. GM electrodes were fixed lengthwise over the centre of the muscle belly. TA electrodes were placed on the muscle belly parallel to the longitudinal axis of the muscle, one‐third of the distance between the head of the fibula and the tip of the medial malleolus. Before fixing the electrodes, the skin was shaved, gently abraded and then cleaned with alcohol. The common reference electrode was placed centrally on the same leg (between the stimulation and recording sites). The locations of the electrodes were documented via photography in order to facilitate their replacement in the same position during the second test session (i.e. post‐test). Signals were amplified (1000×) with a bio‐amplifier g.BSamp 0201a (Guger Technologies, Shieldberg, Austria) and bandpass filtered (5–500 Hz). The signals were acquired on a personal computer at a sampling rate of 2 kHz with a data‐acquisition system (CED power 1401‐3A) and analysed off‐line with Signal 7 software.

#### 2.5.3 Neural stimulation

Stimulations were delivered at an ankle angle of 0° using the CED acquisition system. During eccentric contractions, stimulations were automatically triggered when the ankle angle passed 0°.

#### 2.5.4 Transcranial magnetic stimulation

Single and paired pulse transcranial magnetic stimulations (TMSs) were elicited to assess corticospinal excitability and short‐interval intracortical inhibition (SICI). Motor evoked potentials (MEPs) were elicited in SOL and GM muscles by two Magstim 200^2^ stimulators (Magstim Co, Dyfed, UK) connected via a Bistim^2^ unit and a concave 110‐mm double‐cone coil. The coil was positioned over the primary motor cortex on the side contralateral to the right leg (i.e. left brain hemisphere). Its orientation made it possible to generate posterior‐to‐anterior cortical current, as recommended by Mills and Schubert (1995). It is worth noting that, while differences in corticospinal responses between the dominant and non‐dominant hemispheres have been observed in upper limb studies, there is currently a lack of evidence to suggest a similar disparity in lower limb responses (Smith et al., 2017). Each participant was equipped with a personal snug‐fitted cap (EasyCap, Wörthsee, Germany). In the first session, lines were drawn on the snug‐fitted cap between the preauricular points and from nasion to inion to identify the vertex. In the second session, the same snug‐fitted cap with the drawn lines was replaced at the same location to ensure consistency of data collection across testing sessions (Latella et al., 2016). The central point of the coil was positioned 1 cm lateral and posterior to the vertex, following the approach described by Devanne et al. (1997). Subsequently, the coil position was meticulously adjusted in small medio‐lateral and posterior–anterior steps around the initial position. At each step, a stimulus was delivered at 60% maximal stimulator output during brief isometric voluntary contraction of the plantar flexors at 50% maximal EMG level (EMG_max_) of the SOL (further details in the ‘Visual EMG biofeedback’ section) the location consistently eliciting the greater amplitude for SOL MEP (i.e. the hotspot) was found. Once identified, the front as well as the back of the coil was marked directly on the snug‐fitted cap to ensure consistent positioning throughout the trial and for later testing (Kidgell et al., 2015; Leung et al., 2015). The transcranial active motor threshold was subsequently determined with the ankle at a 0° angle (anatomical zero) during both isometric and eccentric contractions at 50% SOL EMG_max_. As demonstrated by Duclay et al. (2014), modulations in both MEP and H reflex responses remained consistent across different contraction types, irrespective of whether the feedback was based on 50% of SOL EMG_max_ or 50% of maximal voluntary contraction torque.

To initiate the assessment of the transcranial motor threshold, participants were subjected to TMS at intensities below the threshold, starting at approximately 30% of the maximum stimulator output. Subsequently, the stimulus intensity was systematically increased in 5% increments until TMS consistently elicited MEP amplitudes defined as the intensity at which 5 of 10 evoked responses were detected above background EMG levels (Sacco et al., 1997). Following this stage, the stimulus intensity was methodically reduced by 1% increments until fewer than 5 out of 10 responses corresponding to amplitude responses were detected above background EMG levels (Sacco et al., 1997). This specific stimulus intensity, increased by 1% of the stimulator output, was then considered as the active motor threshold intensity.

#### 2.5.5 Percutaneous electrical nerve stimulation

The study of the H reflex elicited by percutaneous electrical nerve stimulation was used to estimate effectiveness of Ia afferents to discharge α‐motoneurons (Aagaard et al., 2002). H reflex and direct motor response (M wave) in SOL and GM were elicited through stimulations of the posterior tibial nerve. This stimulation was achieved using a single rectangular pulse lasting 1 ms, delivered by a stimulator (model DS8R Biphasic Constant Current Stimulator; Digitimer, Welwyn Garden City, UK). To deliver the stimulation, a self‐adhesive electrode with a diameter of 1 cm, composed of Ag–AgCl, served as the cathode and was positioned in the popliteal fossa. The anode electrode (5 × 10 cm, Medicompex SA, Ecublens, Switzerland) was placed on the anterior surface of the knee, situated below the patella. To locate the optimal stimulation site that produced the largest response amplitude, a cathode ball electrode with a diameter of 0.5 cm was initially hand‐held. Once the optimal position was identified, the cathode electrode was securely fixed to this site using straps and tape, following established recommendations (Cattagni et al., 2018). To examine post‐activation depression by primary afferent depolarization (PAD), we employed the D1 method (antagonist afferent conditioning), stimulating the fibular nerve with a second stimulator (Digitimer model DS7R Current Stimulator) by positioning the cathode electrode near the head of the fibula and placing the anode electrode in the vicinity of the medial portion of the tibial head (Colard et al., 2023; Mizuno et al., 1971). The motor threshold for fibular nerve stimulation was determined as the lowest intensity level capable of evoking at least three M waves out of five consecutive stimulations (further details in the ‘Post‐activation depression by PAD’ section).

### 2.6 Experimental protocol

**FIGURE 1:**
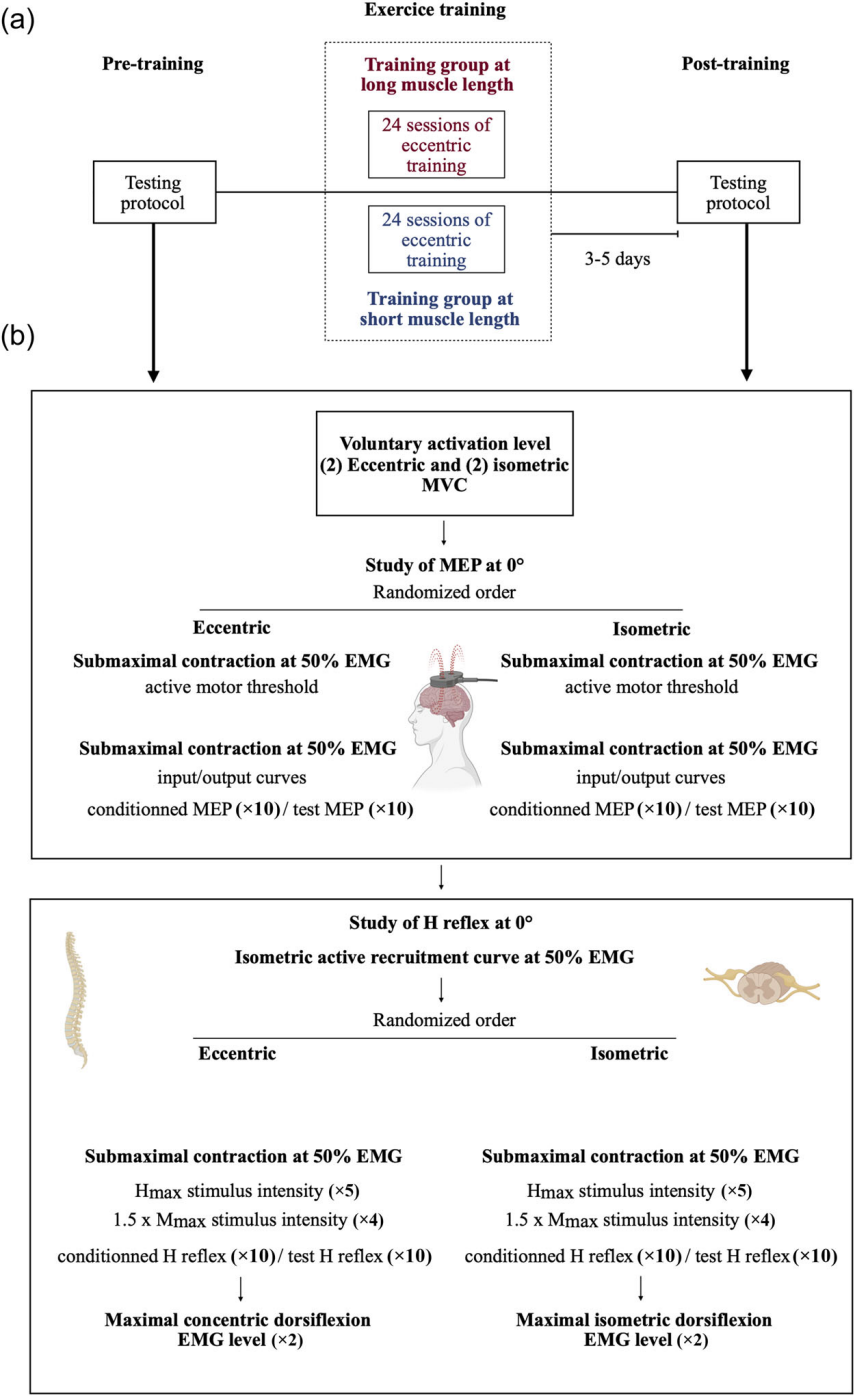
Experimental protocol. (a) General experimental protocol. (b) Neurophysiological assessments. EMG, surface electromyography; H_max_, maximal H reflex; MEP, motor evoked potential; M_max_, maximal M wave amplitude; MVC, maximal voluntary contraction.

Figure 1 summarizes the experimental protocol.

#### 2.6.1 Assessment of maximal neuromuscular performance

Each experimental session started with a standardized warm‐up protocol in which participants performed 10 × 5 s isometric contractions at approximately 50%, 60%, 70% and 80% of maximal voluntary contraction (MVC) with 1 min rest period between each. After a 5‐min rest period, the assessments were made of plantar flexor MVC during isometric and eccentric contractions. The order between isometric and eccentric assessments was randomized and these were separated by a 5‐min rest period.

For the isometric assessment, participants performed two 5‐s plantar flexor isometric MVCs (including 1 s of preactivation and 1 s rest at the end) separated by a 2‐min rest period. Paired supramaximal stimuli (100 Hz) were delivered during (superimposed doublet) and 3 s after (potentiated doublet) the MVC to investigate plantar flexor voluntary activation level (VAL) using the twitch interpolation technique (Strojnik & Komi, 1998). For the eccentric assessment, participants performed 5‐s plantar flexor eccentric MVCs (including 1 s of preactivation and 1 s of rest to return to the initial position) separated by a 2‐min rest period. Participants received the same verbal encouragement in each condition. Finally, participants performed two isometric and two concentric MVCs of dorsiflexors separated by a 2‐min rest period. These contractions were used to determine the magnitude of antagonist TA coactivation (Duclay et al., 2011).

#### 2.6.2 Visual EMG biofeedback

The maximum SOL EMG activity recorded during the pre‐ and post‐training session during maximal isometric MVC was used to determine the target activation level (50% of EMG_max_) to be applied to participants during the two testing sessions (relative matching). In these sessions, participants were asked to contract their plantar flexors so that they moved and maintained their SOL EMG biofeedback on the target, which corresponded to 50% EMG_max_. The SOL EMG biofeedback corresponded to the root mean square (RMS) value of the EMG signal provided by a digital computing channel. This channel instantaneously computed the RMS level of the amplified EMG signal with an integration time of 500 ms. This method allowed muscle activation levels to be maintained constant between all sessions (Duclay et al., 2014).

#### 2.6.3 Motor evoked potentials by transcranial magnetic stimulation

The motor evoked potential (MEP) represents the net excitability of the entire motor pathway, including the primary motor cortex, the corticospinal tract, the spinal cord and α‐motoneurons. For ease of reading, we will use the expression ‘corticospinal excitability’. To investigate corticospinal excitability, input–output curves (Devanne et al., 1997) for MEP in both SOL and GM were determined when participants performed eccentric and isometric submaximal contractions. The stimulus intensity was increased in steps of 10% of the active motor threshold intensity (from threshold 100% to an intensity corresponding at 170% of active motor threshold intensity according to participants). For each stimulus intensity, participants were asked to perform five eccentric and isometric contractions. For eccentric contractions, each trial began with an isometric preactivation of 1 s corresponding to 50% of maximal EMG. The participants were instructed to maintain this level of EMG activity throughout the whole range of motion. The total duration of one movement cycle lasted 5 s, including the 1‐s isometric preactivation period, the 3‐s eccentric contraction (from the initial to the terminal position), and the 1‐s period to return to the initial position. This time duration corresponded to the duration of contraction during isometric testing. Stimulations were always delivered at 0° (neutral position) in both eccentric and isometric contractions.

#### 2.6.4 Short‐interval intracortical inhibition

Short interval intracortical inhibition (SICI) was evaluated using paired‐pulse TMS during eccentric and isometric submaximal (50%) contractions in SOL. When a subthreshold conditioning stimulus precedes a suprathreshold test stimulus by a short interval (less than 5 ms), it leads to a decrease in MEPs (inhibition) compared with a single strong stimulus (Kidgell et al., 2017). This conditioning stimulus triggers low‐threshold inhibitory circuits mediated by GABAergic inhibitory neurons acting via GABA_A_ receptors (Kujirai et al., 1993; Vucic et al., 2009), causing a decrease in activity in the targeted corticospinal cells (Müller‐Dahlhaus et al., 2008). The ratio between paired‐pulse and single‐pulse MEP amplitudes reflects SICI. To quantify SICI, 10 conditioned stimuli (paired pulses) and 10 non‐conditioned stimuli (a single test pulse only) were delivered in a random order (Kidgell et al., 2015) for each contraction type (eccentric and isometric). The stimulator output intensity was set at 120% of the active motor threshold (which was determined via the input–output curves) (Brownstein et al., 2018; Neige et al., 2020; Škarabot et al., 2019). The conditioning stimulus for paired‐pulse stimulation was set at 70% of the active motor threshold and the interstimulus interval (ISI) was 2 ms (Brownstein et al., 2018).

#### 2.6.5 Potentials evoked by peripheral nerve stimulation

H reflex and M wave recruitment curves from SOL and GM were first obtained during isometric voluntary contraction at 50% of SOL EMG_max_. The stimulation intensity was progressively increased in 2 mA increments, starting from the H reflex threshold, until the intensity at which no further changes in the amplitude of the M wave (i.e. plateau) were observed. Five stimulations were delivered at each stimulation intensity, using an interstimulus interval of 5 s. This number of stimulations, that is, the evoked H reflex, is sufficient to obtain reliable data (Hopkins et al., 2000; Theodosiadou et al., 2023). To record SOL and GM H_max_, and M_max_ values during isometric and eccentric submaximal (50% EMG_max_) contractions, five stimulations at H_max_ and four stimulations at M_max_ intensity (intensities defined during isometric recruitment curves) were delivered for each condition. The cycle of movements and angle of stimulations were exactly the same as for the study of MEPs.

#### 2.6.6 Post‐activation depression by primary afferent depolarization

To investigate the presynaptic mechanism at the level of the Ia‐α‐motoneuron synapse, we studied post‐activation depression by primary afferent depolarization (PAD) (Metz et al., 2023). To do this, we used the D1 method, as described by Mizuno et al. (1971). To evoke SOL‐conditioned H reflex (H_D1_), a train of three 1‐ms stimulations at 300 Hz was applied to the fibular nerve, with an intensity equivalent to 1.2 times the stimulation intensity of the tibialis anterior motor threshold. The interval between the first shock of the train and the SOL H_test_ was 21 ms (Aymard et al., 2000; Colard et al., 2023; Lamy et al., 2009). The intensity selected to elicit H_test_ was comparable to that which produced H_max_. A total of 20 SOL H_test_ and 20 SOL H_D1_ were randomly evoked for both eccentric and isometric contractions.

### 2.7 Data analysis

#### 2.7.1 Muscle torque and voluntary activation

For the plantar flexor muscles, MVC torque was considered as the highest peak torque value measured over three trials. VAL was quantified by measuring the amplitudes of the superimposed doublet twitch peak torque and the potentiated doublet twitch peak torque from the MVC trial with the highest peak torque. VAL was estimated according to the following formula, including the Strojnik & Komi (1998) correction:

$$\begin{array}{c} \mathrm{VAL}\;=\left[1-\frac{\mathrm{Superimposed}\;\mathrm{doublet}\;\mathrm{twitch}\;\mathrm{torque}\;\times \;\frac{\mathrm{Torque}\;\mathrm{at}\;\mathrm{stimulation}\;}{\mathrm{MVC}\;\mathrm{torque}}}{\mathrm{Potentiated}\;\mathrm{doublet}\;\mathrm{twitch}\;\mathrm{torque}}\right]\\ \;\times \;100 \end{array}$$

#### 2.7.2 EMG activity

The quantification of SOL and GM EMG activity was calculated by the RMS value during the 500‐ms window preceding the stimulation. This value was subsequently normalized to the corresponding maximal M wave value (EMG_RMS_/M_max_). Similarly, the RMS value of the tibialis anterior EMG (tibialis anterior EMG_RMS_) was also analysed over the same time period preceding the stimulation. In addition, we normalized the EMG_RMS_ value by the maximal EMG_RMS_ level (EMG_RMS_/EMG_RMSmax_) in order to ensure that any differences found between groups were not a result of differences in pre‐stimulus EMG activity. To quantify the level of coactivation during isometric contractions, tibialis anterior EMG_RMS_ was expressed as a fraction of its maximal value obtained for the isometric MVC of the dorsiflexors. To quantify coactivation during eccentric contractions, tibialis anterior EMG_RMS_ obtained during lengthening plantar flexions was normalized to concentric dorsiflexion MVC (Duclay et al., 2011, 2014).

#### 2.7.3 Motor evoked potentials

Input–output curves were generated from MEP responses recorded in both the SOL and GM when subjects performed submaximal eccentric and isometric contractions (50% EMG_max_). For each muscle, the peak‐to‐peak amplitude of MEPs was measured. For each contraction type, the size of the SOL and GM MEP was normalized to the corresponding M wave obtained in the same condition (MEP_max_/M_max_). The following equation of Boltzmann's sigmoidal function was used to fit the data points (Carroll et al., 2002; Devanne et al., 1997):

$$\mathrm{ME}P_{\left(s\right)}\;=\mathrm{ME}P_{\max}\;\times \frac{1}{\frac{\left(S_{50}-S\right)}{1+e^{\mathrm{ME}P_{\mathrm{slope}}}}}$$

where MEP_max_ is the maximum MEP defined by the function, *S* is the stimulus intensity and *S*_50_ is the stimulus intensity at which the MEP size is 50% of the maximal MEP. MEP_slope_ was calculated by differentiating the input–output equation. As suggested previously (Devanne et al., 1997), these parameters need to be assessed to investigate a task‐dependent changes in corticospinal excitability.

To quantify SICI, peak‐to‐peak amplitudes of conditioned and non‐conditioned SOL MEPs were calculated. Given the dependency of SICI on the number of activated α‐motoneurons at the spinal level (Lackmy & Marchand‐Pauvert, 2010) and the fact that intracortical inhibition becomes more pronounced with greater spinal motoneuron recruitment, we used the SICI_Mmax_ equation (Lackmy & Marchand‐Pauvert, 2010; Neige et al., 2020). This equation was used to calculate the difference between the mean MEP_cond_ and the mean MEP_test_ then expressed as a percentage of the mean M_max_:

$$\mathrm{SIC}I_{\mathrm{Mmax}}=\frac{\left(\mathrm{ME}P_{\mathrm{cond}}-\mathrm{ME}P_{\mathrm{test}}\right)}{M_{\max}}\;\times \;100$$

where MEP_cond_ was the conditioned MEP and MEP_test_ the non‐conditioned MEP. The equation gives an indication of the number of α‐motoneurons actives across the two experimental sessions. Additionally, since the peak amplitude of the test response can influence SICI outcomes (Lackmy‐Vallee et al., 2012), we normalized the MEP_test_ offline to the corresponding M_max_ values (MEP_test_/M_max_ ratio). This ratio enabled us to verify that the MEP_test_ amplitude normalized to M_max_ remained constant across test.

#### 2.7.4 H reflex and M wave

The average values of H_max_, M_max_, H_D1_ and H_test_ were calculated from peak‐to‐peak amplitudes. To estimate the effectiveness of activated Ia afferents to discharge α‐motoneurons (Theodosiadou et al., 2023), we calculated the H_max_/M_max_ ratio. The SOL and GM M waves elicited concomitantly with the H_max_ (M_at_H_max_), which represents a small fraction of the M_max_, was measured and the average value for each corresponding condition was analysed. To examine post activation by PAD through the D1 method, we calculated the SOL H_D1_/H_test_ ratio, that is, the further the ratio is from 1, the greater the post‐activation depression by PAD. We also calculated the SOL H_test_/M_max_ ratio to determine whether the amplitude of the SOL H_test_ was constant between the experimental sessions. This enabled us to check that we had the same sensitivity from excitatory and inhibitory inputs between the different conditions (Baudry & Duchateau, 2012).

#### 2.7.5 Statistical analysis

**FIGURE 2:**
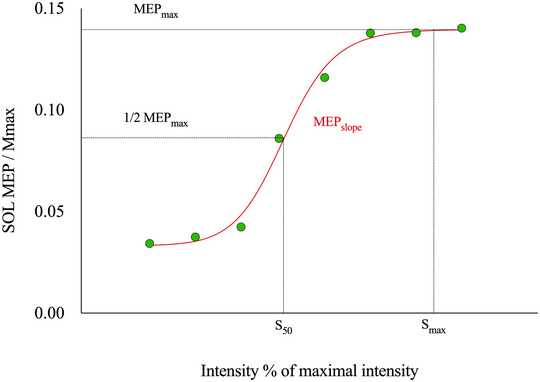
An example of a Boltzmann fit to MEP size versus stimulus intensity plot. MEP_max_, maximal motor evoked potential; *S*_50_, the stimulus intensity at which the MEP size is 50% of the maximal MEP; *S*_max_, the stimulation intensity at which the MEP size is the maximal MEP.

All descriptive statistics presented in the text and figures are given as mean values ± standard deviation. The significance level was set at *P* < 0.05 for all analyses. Data normality was verified using the Shapiro–Wilk normality test and sphericity was assumed. Three‐factor [training group (LONgroup vs. SHOgroup) × testing session (pre‐training vs. post‐training) × contraction type (eccentric vs. isometric)] ANOVAs with repeated measures on training and contraction type were used to compare MVC torque, VAL, SICI_Mmax_, MEP_test_/M_max_, H_D1_/H_test_, H_test_/M_max_ and TA coactivation. Four‐factor [training group (LONgroup vs. SHOgroup) × testing session (pre‐training vs. post‐training) × contraction type (eccentric vs. isometric) × muscle (SOL vs. GM)] ANOVAs with repeated measures on training, contraction type and muscle were used to compare 100%EMG_RMS_/M_max_, 50%EMG_RMS_/M_max_, EMG_RMS_/EMG_RMSmax_, MEP_max_/M_max_, MEP_slope_, maximal M wave amplitude (M_max_), H_max_/M_max_ and M_at_H_max_/M_max_. Whenever a significant main effect or interaction was detected, Tukey's correction was performed for *post hoc* analysis. These statistical analyses were performed using JASP (Version 0.17.2.1, NED) and GraphPad Prism software (version 9.0; GraphPad Software, Boston, MA, USA) (Figure 2).

## 3 RESULTS

For clarity, to restrict the scope of our analysis and focus on the hypotheses of this study, namely the interaction between training group effect and testing session effect, we present the results involving the main factors ‘training group’ (LONgroup vs. SHOgroup) and ‘testing session’ (pre‐training vs. post‐training) and the interactions including at least these two factors (ANOVA results are presented in Supporting information, Table A1). Note that no significant baseline differences were observed between the two participant groups for any of the dependent variables.

### 3.1 Neuromuscular performance and activation

#### 3.1.1 Maximal voluntary contraction torque

**FIGURE 3:**
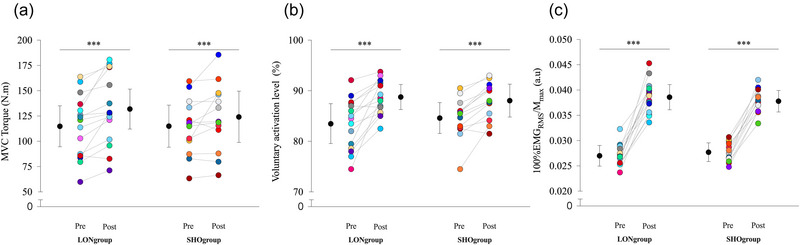
Changes in maximal voluntary torque, maximal voluntary activation and 100%EMG level before and after 9 weeks of eccentric training. Absolute and individual data (*n* = 28) are expressed as means ± standard deviation. Maximal plantar flexion torque (a), maximal voluntary activation (b) and normalized EMG_RMS_ activity (c) at pre‐training and post‐training. ***Significant difference at *P* < 0.001 for training effect: pre‐training versus post‐training. M_max_, maximal M wave amplitude; MVC, maximal voluntary contraction; RMS, root mean square.

Maximal voluntary contraction torque (*T*_max_) was calculated to examine plantar flexor strength. Repeated measures ANOVA showed an effect of testing session (*P* < 0.001). *Post hoc* analysis showed that *T*_max_ was 12.66% (*P* < 0.001) greater post‐training than pre‐training, regardless of training group (Figure 3a).

#### 3.1.2 Estimates of neuromuscular activation during maximal voluntary contraction

VAL and plantar flexor 100%EMG_RMS_/M_max_ were calculated to estimate the level of neuromuscular activation during MVC. Repeated measures ANOVA for VAL showed an effect of testing session (*P* < 0.001). *Post hoc* analysis showed that VAL was 5.3% (*P* < 0.001) greater post‐training than pre‐training, regardless of training group (Figure 3b).

For SOL and GM 100%EMG_RMS_/M_max_ and 50%EMG_RMS_/M_max_, repeated measures ANOVA showed an effect of testing session (*P* < 0.001). *Post hoc* analysis showed that plantar flexor 100%EMG_RMS_/M_max_ and 50%EMG_RMS_/M_max_ was 40.2% (*P* < 0.001, Figure 3c) and 39,8% (*P* < 0.001) greater post‐training than pre‐training, respectively, regardless of training group. No significant modifications of EMG_RMS_/EMG_RMSmax_ and TA coactivation were observed before or after training (all *P* values >0.652 and 0.279, respectively; Supporting information, Table A2).

### 3.2 Motor evoked potential (MEP)

#### 3.2.1 Estimates of corticospinal excitability

**FIGURE 4:**
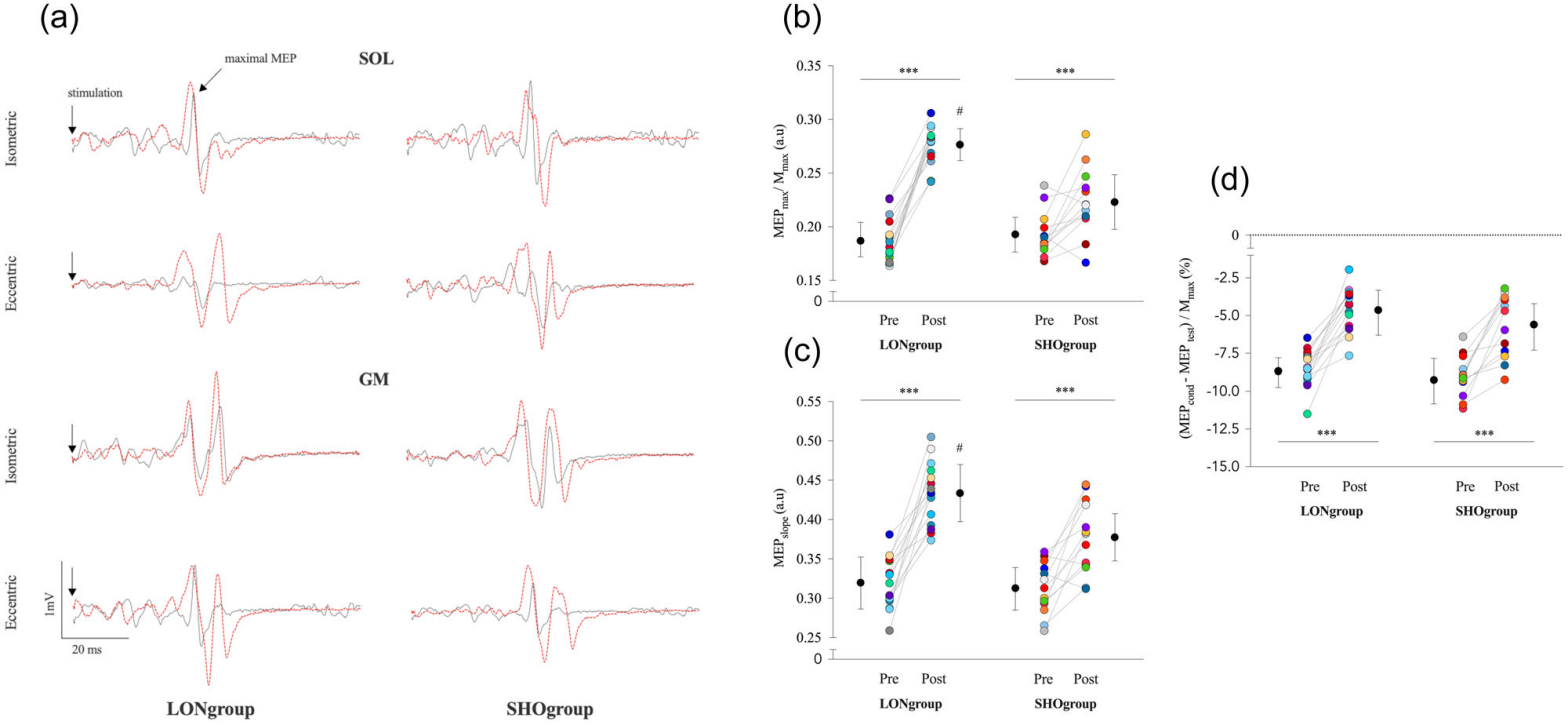
Changes in corticospinal and cortical parameters before and after 9 weeks of eccentric training. (a) Representative traces showing the maximal motor evoked potentials recorded during isometric and eccentric submaximal contractions of soleus and gastrocnemius medialis for one representative subject of each muscle training group (long muscle length group vs. short muscle length group) before (black lines) and after (red lines) 9 weeks of eccentric training. Absolute and individual data (*n* = 28) are expressed as means ± standard deviation. (b) Modulations of the MEP_max_/M_max_ ratio are represented for both training groups (LONgroup and SHOgroup) during the pre‐training and post‐training. (c, d) Modulations of MEP_slope_ (c) and SOL SICI_Mmax_ ((MEP_cond—_MEP_test_)/M_max_) (d). #Significant difference at *P* < 0.001 for training group effect: post LONgroup versus post SHOgroup. ***Significant difference at *P* < 0.001 for testing session effect: pre‐training versus post‐training. SICI, short‐interval intracortical inhibition.

Figures 4a illustrate representative data from the assessment of corticospinal excitability and raw traces showing SOL and GM MEP_max_ in one individual from each muscle training group. For MEP_max_/M_max_, repeated measures ANOVA showed an effect of training group (*P* < 0.001), testing session (*P* < 0.001) and an interaction of training group × testing session (*P* < 0.001). *Post hoc* analysis showed that MEP_max_/M_max_ was 47.1% and 15.7% (*P* < 0.001) greater post‐training compared with pre‐training for the LONgroup and SHOgroup, respectively. MEP_max_/M_max_ was 24.03% (*P* < 0.001) greater for the LONgroup compared with SHOgroup post‐training (Figure 4b).

For MEP_slope_, repeated measures ANOVA revealed effects of training group (*P* = 0.007) and testing session (*P* < 0.001) and an interaction of training group × testing session (*P* = 0.016). *Post hoc* analysis showed that MEP_slope_ was 35.5% and 20.7% (*P* < 0.001) greater post‐training compared with pre‐training for the LONgroup and SHOgroup, respectively. MEP_slope_ was 14.8% (*P* < 0.001) greater for the LONgroup compared with SHOgroup post‐training (Figure 4c).

#### 3.2.2 Estimates of cortical inhibition within the primary motor cortex (M1)

For SICI_Mmax_, repeated measures ANOVA showed an effect of testing session (*P* < 0.001). *Post hoc* analysis showed that SICI_Mmax_ was 43.1% (*P* < 0.001) reduced post‐training than pre‐training, regardless of training group (Figure 4d).

For MEP_test_/M_max_, repeated measures ANOVA showed no effect or interaction (all *P* > 0.115), indicating that SOL MEP_test_/M_max_ did not differ significantly between the training groups before or after the intervention.

### 3.3 Potentials evoked by peripheral nerve stimulation

#### 3.3.1 Estimates of effectiveness of activated Ia afferents to discharge α‐motoneurons

**FIGURE 5:**
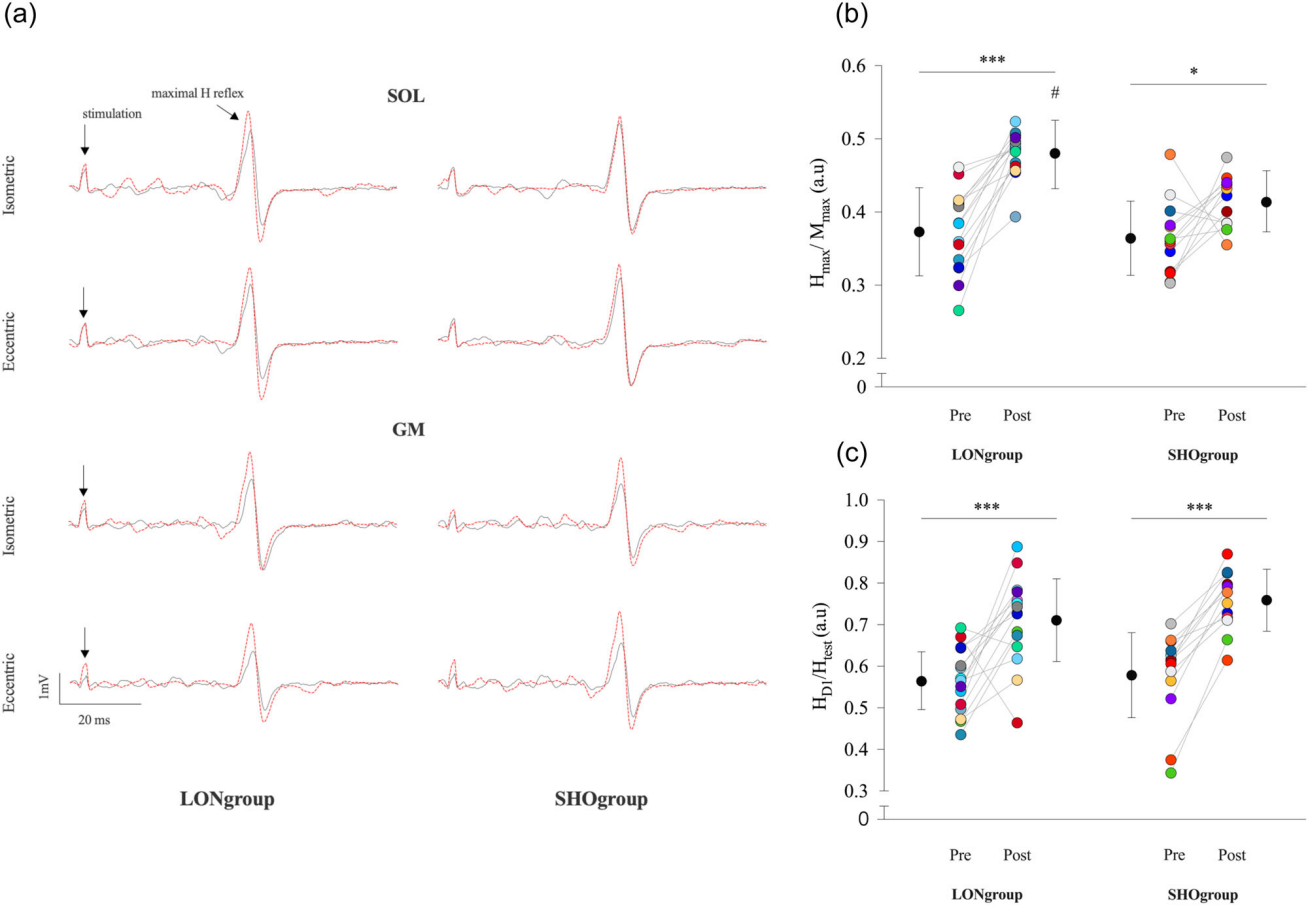
Changes in effectiveness of activated Ia afferents to discharge α‐motoneurons and post‐activation depression by primary afferent depolarization before and after 9 weeks of eccentric training. (a) Representative traces showing soleus and gastrocnemius medialis maximal H reflex evoked by posterior tibial nerve stimulation before (black lines) and after (red lines) 9 weeks of eccentric training. Absolute and individual data (*n* = 28) are expressed as means ± standard deviation. (b, c) Modulations of the H_max_/M_max_ (b) and SOL H_D1_/H_test_ (c) are represented for both training groups (LONgroup and SHOgroup). #Significant difference at *P* < 0.001 for training group effect: post LONgroup versus post SHOgroup. *Significant difference at *P* < 0.05 for testing session effect pre‐training versus post‐training. ***Significant difference at *P* < 0.001 for testing session effect: pre‐training versus post‐training.

Figure 5a illustrates raw traces showing the SOL and GM H reflex for one individual from each muscle training group. For H_max_/M_max_, repeated measures ANOVA showed an effect of training group (*P* = 0.002), an effect of testing session (*P* < 0.001), and an interaction of training group × testing session (*P* = 0.030). *Post hoc* analysis showed that H_max_/M_max_ was 28.7% and 13.5% (*P* < 0.001 and *P* = 0.038) greater post‐training than pre‐training for LONgroup and SHOgroup, respectively (Figure 5b). The increase of H_max_/M_max_ after training was 16.1% (*P* < 0.001) greater for LONgroup than for SHOgroup. These results show that the effectiveness of activated Ia afferents to discharge α‐motoneurons increased more significantly in LONgroup than in SHOgroup.

Note that the recording conditions were the same between training groups and testing session since no main effect or interaction was found for M_max_ (all *P* > 0.067). Moreover, as repeated measures ANOVA showed no main effect or interaction for the M_at_H_max_/M_max_ (*P* = 0.344), this indicates that neither M_max_ nor M_at_H_max_/M_max_ was different between sessions (Supporting information, Table A2).

#### 3.3.2 Post‐activation depression by PAD

For SOL H_D1_/H_test_, repeated measures ANOVA only showed an effect of testing session (*P* > 0.001). *Post hoc* analysis showed that SOL H_D1_/H_test_ ratio was 28.2% (*P* < 0.001) greater post‐training compared with pre‐training, regardless of training group (Figure 5c). Note that the amplitude of H_test_ was constant between training groups and testing session since no main effect or interaction was found for H_test_/M_max_ (all *P* > 0.344; Supporting information, Table A2).

## 4 DISCUSSION

The present study examined for the first time whether eccentric training at different muscle lengths leads to different neuromuscular adaptations. Before and after the training period, we used different electrophysiological methods to provide experimental estimates of adaptations at different levels of the central nervous system. We showed that eccentric training at short and long plantar flexor lengths promoted neural plasticity leading to neuromuscular activation and muscle strength gains. Interestingly, in accordance with our hypothesis, eccentric training induced greater corticospinal excitability and spinal reflex adaptations when performed at long muscle length. Notably, corticospinal excitability and effectiveness of activated Ia afferents to discharge α‐motoneurons during contraction showed a more pronounced increase after eccentric training at long than at short muscle lengths. However, these differences in neurophysiological adaptations did not result in different neuromuscular activation and strength gains. The present findings support the consideration of muscle length in eccentric training as this is important to promote neural plasticity even though it does not seem to impact strength gains.

### 4.1 Corticospinal and cortical adaptations

Results for MEP/M_max_ and MEP_slope_ show that eccentric training influences the regulation of corticospinal excitability in plantar flexors. This confirms recent reports showing that corticospinal excitability is significantly increased after eccentric training (Kidgell et al., 2017; Latella et al., 2019; Siddique et al., 2020; Tallent et al., 2021). Our original finding is that the amplitude of this adaptation depends on the length at which muscles are trained. Indeed, we found that plantar flexor MEP_max_/M_max_ was more pronounced after eccentric training at long muscle length than after eccentric training at short muscle length.

A potential explanation for the greater neural adaptation following eccentric training at greater muscle length compared to short length could be the greater sensory feedback related to the difficulty of this type of exercise. For example, numerous studies have shown that in motor learning situations, greater attention and sensory input lead to task‐specific changes in the neural circuits of the M1 (Carey et al., 2006; Jensen et al., 2005; Leung et al., 2015). Given the similarity between long‐length eccentric training and motor learning, in the early stages of skill acquisition, the processing of new sensory signals with the correct motor commands is critically dependent on afferent feedback (Halsband & Lange, 2006). Furthermore, dynamic contractions have been shown to increase sensory afferent feedback to supraspinal regions (Dimitriou, 2022; Gandevia & Burke, 1990). Consequently, it is plausible that the continuous sensory feedback resulting from eccentric training at long muscle length causes repeated sensory input to the M1, potentially modulating corticospinal and cortical inhibitions.

Nevertheless, although we observed a decrease in the responsiveness of intracortical inhibitory interneurons (SICI) to TMS after eccentric training, we did not find any effect of the muscle length at which muscles were trained. Muscle contraction has been shown to enhance sensory feedback from Ia afferents, leading to a reduction in intracortical inhibition (Brerro‐Saby et al., 2008; Vie et al., 2013). Consequently, the observed result is unexpected, as Ia afferent discharge should be greater during eccentric training at a long muscle length than at a short muscle length. While cortical inhibitory mechanisms alone cannot explain the greater increase in corticospinal excitability after long‐term eccentric training, other neural adjustments at spinal level could potentially be the most relevant explanation in this specific adaptation (Škarabot et al., 2019; Yacyshyn et al., 2016).

### 4.2 Effectiveness of activated Ia afferents to discharge α‐motoneurons

As previously observed, our results on H_max_/M_max_ show an increase in the effectiveness of activated Ia afferents to discharge α‐motoneurons following eccentric training in plantar flexors (Duclay et al., 2008). The originality of our study lies in the evidence that the magnitude of adaptations in this effectiveness related to eccentric training is greater when individuals are trained at long plantar flexor lengths compared with short ones. One of the mechanisms known to influence the effectiveness of activated Ia afferents to discharge α‐motoneurons during eccentric contraction is post‐activation depression by PAD (Colard et al., 2023; Papitsa et al., 2022). The mechanism involves PAD‐evoked spikes that lead to a decrease in neurotransmitter release at the Ia afferent terminal (Hari et al., 2022). In this study, we tested for the first time whether post‐activation depression by PAD adapts to eccentric training. Interestingly, our findings demonstrate that post‐activation depression by PAD can be reduced by eccentric exercise, as shown by the increase of H_D1_/H_test_ after the eccentric training in both training groups. One plausible explanation lies in the chronic exposure to the mechanical constraints imposed by the eccentric contraction type, which may produce a substantial change in supraspinal command to the GABAergic interneurons (Grosprêtre et al., 2014). Consequently, these interneurons could experience saturation of their activities, which is known as the occlusion phenomenon (Pierrot‐Deseilligny & Burke, 2005). In response to such alterations, it is likely that supraspinal command could reverse the process, raising the excitability threshold of these GABAergic interneurons. This adjustment could make them less receptive to inputs from afferent collaterals, thereby decreasing post‐activation depression. However, H_D1_/H_test_ increased similarly for both training groups, indicating that adaptation of post‐activation depression by PAD related to eccentric training is not influenced by the muscle length at which plantar flexors are trained.

### 4.3 Potential post‐synaptic adaptations

Taken together (i) the greater increase of corticospinal excitability and higher effectiveness of Ia afferents to discharge α‐motoneurons after eccentric training at a long muscle length compared with a short one, and (ii) the similar adaptation of post‐activation depression by PAD between these interventions suggest that postsynaptic mechanisms modulating α‐motoneuron excitability may be the most likely elements contributing to the neurophysiological adaptations specific to the length at which the muscles were trained.

Recurrent inhibition may be a relevant candidate for this specific adaptation (Katz & Pierrot‐Deseilligny, 1999; Pierrot‐Deseilligny & Burke, 2005), as it directly alters the excitability of α‐motoneurons through the activity of Renshaw cells. In fact, a recent study showed that the level of recurrent inhibition, during eccentric contraction was higher at long muscle length than at short muscle length (Colard et al., 2024) reflecting a specific control of this inhibition in these conditions. Since inhibition is greater during eccentric contractions at long muscle length than at shorter length, prolonged exposure to such contractions may lead to a greater release of this inhibition over training. This theory is supported by the findings of Duclay et al. (2008), which demonstrate that after eccentric training, the inhibition of the H reflex typically observed is reduced (i.e. prolonged exposure to this inhibition). Another possible explanation involves changes in reciprocal inhibition mediated by antagonist muscle. For instance, studies have shown that a reduction in reciprocal inhibition from antagonist muscle may result in an increase in agonist α‐motoneurons activity (Hultborn et al., 1976; Mizuno et al., 1971). It is therefore plausible that changes in reciprocal inhibition from antagonist muscle affecting the agonist muscle contribute to the enhanced spinal H reflex and corticospinal excitability observed following eccentric training at long muscle length. Our observations did not indicate any notable change in the coactivation of the tibialis anterior in either training group prior to or following training. In light of the above, we can conclude that reciprocal inhibition has a limited and non‐dominant effect on the neurophysiological adaptations specific to the length at which the muscles were trained.

Training at long versus short muscle length may also induce different adaptations of persistent inward currents (PICs) acting on α‐motoneurons. PICs regulate the intrinsic properties of α‐motoneurons, with their amplitude influenced by both inhibitory and neuromodulatory inputs (Bui et al., 2008; Hyngstrom et al., 2007). Recent findings showed that resistance training may enhance PICs (Martino et al., 2024; Orssatto et al., 2023). Although our eccentric training may have modulated PICs, it is unlikely that different muscle lengths lead to different adaptations in neuromodulatory inputs. However, since PICs may be potentially shaped by inhibitory inputs, a decreased inhibition resulting from eccentric training at longer muscle length may have enhanced PIC magnitude.

### 4.4 Neuromuscular adaptations and strength gains

The present study confirms that eccentric training leads to neuromuscular activation and strength gains. Surprisingly, despite the difference in neurophysiological adaptations between the training groups, we did not observe different amplitudes of neuromuscular activation or strength gains between eccentric training at long versus short plantar flexor lengths.

In agreement with previous studies, we observed increases in maximal torque, muscle activity (Duclay et al., 2008) and voluntary activation level (Maffiuletti et al., 2002) after eccentric training of the plantar flexors. This suggests that eccentric training improves the neural drive to trained muscles, regardless of the muscle length at which plantar flexors are trained. The absence of any such length effect is surprising because, over a 9‐week training period, we might also have expected an additional strength gain at longer lengths due to architectural adaptations of the plantar flexors.

### 4.5 Methodological considerations

Some methodological factors may explain why we observed (i) no effect of the length at which the muscles were trained, and (ii) a lower strength gain (∼7.2%) after eccentric training compared with previous studies. Firstly, our eccentric training programme mixed two exercise modalities, (i) isotonic (constant resistance) and (ii) isokinetic (same angular velocity), whereas most eccentric training programmes use either only isotonic (Duclay et al., 2008; Ekblom, 2010) or only isokinetic (Barrué‐Belou et al., 2016; Kidgell et al., 2015). Nevertheless, the inclusion of heel raises on a Smith machine and inclined leg press (isotonic exercises) may cause greater mechanical stress to the muscles at the weakest joint angles (Guilhem et al., 2010), that is, at shorter lengths compared with longer ones. Therefore, we cannot exclude the possibility that the few sessions of eccentric training on an isokinetic dynamometer in this study were insufficient to counterbalance this effect. However, the methodological choices regarding the equipment selected for the eccentric training programme aimed to balance experimental control, ecological validity and participant engagement. While isokinetic dynamometry provided optimal precision and standardization, we also incorporated traditional equipment, such as the Smith machine and incline leg press, to better replicate ecological training conditions and enhance the practical relevance of the findings. This approach also helped to improve participant adherence by offering more accessible and engaging exercises, while minimizing logistical constraints.

Secondly, contrary to some previous studies (Duclay et al., 2008; Guex et al., 2016), we did not perform neuromuscular tests at an ankle angle in the range of angles used in the eccentric exercises of the short and long muscle length groups. Studies investigating the impact of eccentric training at long muscle lengths, such as Guex et al. (2016), conducted test measurements under the same conditions (same muscle length) as those used in the training protocol. In the context of our study, we decided to select comparable angles between the two groups in order to characterize a general effect. Consequently, the fact that we performed the test measurements at 0° and not on the corresponding muscle length used in the training may have reduced the sensitivity of our measurements.

We adjusted the training load using the 1RM method, assessed during concentric contractions every two sessions for heel raises on the Smith machine and inclined leg press. The 1RM was used to perform 10 eccentric repetitions for each set, which ensured that the training load (adjusted with 1RM) was sufficient and appropriate to generate muscular strength adaptations (Grgic et al., 2020; Suchomel et al., 2021).

It should be noted that our experimental protocol was not designed to provide information about the time‐course of neural adaptations. It is possible that, despite similar neural drive and strength gains between the groups over the 9 weeks of training, the kinetics of neuromuscular adaptations were different. Additionally, it is worth noting that our study only focused on the plantar flexors because the experimental methods used to investigate neurophysiological adaptations are particularly relevant and validated for this muscle group, especially for the SOL muscle. However, the tendon of this muscle group (Achilles tendon) is known for its specific mechanical properties, particularly its compliance, which gives it good energy dissipative properties (Kubo et al., 2002). Thus, the compliance of the Achilles tendon was able to generate a similar mechanical stress between the training regimes at long and short muscle lengths, without leading to different adaptations. It may explain the small differences in force gain observed between groups. Finally, our sample was optimized to closely match our hypotheses and the anticipated neurophysiological adaptations. This rigorous selection process provided a robust answer to our main research questions. However, it is important to note that such optimization may have unintentionally decreased the sensitivity of the sample to functional variables, potentially limiting the generalizability of our results in this area.

The choice of performing the evoked potential measurements at different contraction levels from the functional variables may have contributed to the discrepancies between adaptations of strengths and evoked potentials. However, this decision was made to limit fatigue and reduce the duration of the experiments. It is therefore possible that the results do not fully reflect the adaptations of the underlying mechanisms or their functional magnitude at 100% of maximal EMG. For performances requiring maximal neuromuscular activity, eccentric training at long muscle lengths does not appear to offer additional functional benefits (i.e. in terms of force) compared to training at short muscle lengths. Conversely, for tasks that do not demand maximal neuromuscular capacity, improved the effectiveness of activated Ia afferents to discharge α‐motoneurons, along with increased corticospinal excitability, could contribute to enhanced performance in these contexts. This could be particularly advantageous for daily activities such as walking, climbing stairs or preventing falls, especially in elderly or pathological populations.

## 5 Conclusion

The findings of this study highlight the importance of considering muscle length during eccentric training to promote neural adaptations, especially at the spinal level. However, they do not support that muscle length is a key parameter in the aim to maximize neuromuscular activation and strength gains during maximal eccentric contractions of the plantar flexors. Further research is needed: (i) to determine whether the same results are found in other muscle groups with less compliant tendons, such as the quadriceps or biceps brachii, and (ii) to determine the mechanisms at the spinal level that are involved in the greater neural adaptation observed when eccentric training is done at long muscle length. For example, investigating the effects of eccentric training at different muscle lengths on recurrent inhibition by Renshaw cells (Barrué‐Belou et al., 2018, 2019) could be a relevant avenue for future study.

## AUTHOR CONTRIBUTIONS

Julian Colard, Marc Jubeau, Antoine Nordez and Thomas Cattagni: conception or design of the work. Julian Colard, Yohan Betus, Marc Jubeau, Tristan Tallio, Baptiste Bizet and Thomas Cattagni: acquisition, analysis or interpretation of data for the work. All authors drafted and revised the work. All authors approved the final version of the manuscript and agree to be accountable for all aspects of the work in ensuring that questions related to the accuracy or integrity of any part of the work are appropriately investigated and resolved. All persons designated as authors qualify for authorship, and all those who qualify for authorship are listed

## CONFLICT OF INTEREST

None declared.

## Supporting information



Tables A1 and A2.

## Data Availability

Individual data are available in the following link: https://doi.org/10.6084/m9.figshare.27053809
