# Supplementary material for: Eccentric training at long muscle lengths induces greater corticospinal and spinal reflex plasticity than eccentric training at short muscle lengths
Source: Exp Physiol. 2025 Jun 2;110(12):1990–2006. doi: 10.1113/EP092470 (PMC12665938; doi:10.1113/EP092470)
Supplement: Supplementary file 1 — Tables A1 and A2. [file EPH-110-1990-s001.docx]

SUPPORTING INFORMATION

| **Measure** | **Training Group** | **Testing session** | **Training group x Testing session** |
| --- | --- | --- | --- |
| **T_max_** | F _(1,26)_ = 0.107; P = 0.747; $\eta_{p}^{2}$= 0.004 | **F _(1,26)_ = 20.401; P < 0.001;** $\boldsymbol{\eta}_{\mathbf{p}}^{\mathbf{2}}$**= 0.440** | F _(1,26)_ = 1.909; P = 0.179; $\eta_{p}^{2}$= 0.068 |
| **VAL** | F _(1,26)_ = 0.020; P = 0.888; $\eta_{p}^{2}$= 7.781×10^−4^ | **F _(1,26)_ = 50.546; P < 0.001;** $\boldsymbol{\eta}_{\mathbf{p}}^{\mathbf{2}}$**= 0.660** | F _(1,26)_ = 2.248; P = 0.146; $\eta_{p}^{2}$= 0.080 |
| **100% EMG_RMS_/M_max_** | F _(1,26)_ = 0.237; P = 0.631; $\eta_{p}^{2}$= 0.009 | **F _(1,26)_ = 356.718; P < 0.001;** $\boldsymbol{\eta}_{\mathbf{p}}^{\mathbf{2}}$**= 0.932** | F _(1,26)_ = 0.011 P = 0.917; $\eta_{p}^{2}$= 4.249×10^−4^ |
| **MEP_max_/M_max_** | **F _(1,26)_ = 12.766; P < 0.001;** $\boldsymbol{\eta}_{\mathbf{p}}^{\mathbf{2}}$**= 0.329** | **F _(1,26)_ = 112.803; P < 0.001;** $\boldsymbol{\eta}_{\mathbf{p}}^{\mathbf{2}}$**= 0.813** | **F _(1,26)_ = 27.658; P < 0.001;** $\boldsymbol{\eta}_{\mathbf{p}}^{\mathbf{2}}$**= 0.515** |
| **MEP_slope_** | **F _(1,26)_ = 8.602; P = 0.007;** $\boldsymbol{\eta}_{\mathbf{p}}^{\mathbf{2}}$**= 0.249** | **F _(1,26)_ = 87.390; P < 0.001;** $\boldsymbol{\eta}_{\mathbf{p}}^{\mathbf{2}}$**= 0.771** | **F _(1,26)_ = 6.575; P = 0.016;** $\boldsymbol{\eta}_{\mathbf{p}}^{\mathbf{2}}$**= 0.202** |
| **SICI_Mmax_** | F _(1,26)_ = 2.160; P = 0.154; $\eta_{p}^{2}$= 0.077 | **F _(1,26)_ = 75.079; P < 0.001;** $\boldsymbol{\eta}_{\mathbf{p}}^{\mathbf{2}}$**= 0.743** | F _(1,26)_ = 0.192; P = 0.665; $\eta_{p}^{2}$= 0.007 |
| **H_max_/M_max_** | **F _(1,26)_ = 11.359; P = 0.002;** $\boldsymbol{\eta}_{\mathbf{p}}^{\mathbf{2}}$**= 0.304** | **F _(1,26)_ = 38.740; P < 0.001;** $\boldsymbol{\eta}_{\mathbf{p}}^{\mathbf{2}}$**= 0.598** | **F _(1,26)_ = 5.289; P = 0.030;** $\boldsymbol{\eta}_{\mathbf{p}}^{\mathbf{2}}$**= 0.169** |
| **H_D1_/H_test_** | F _(2,28)_ = 1.435; P = 0.242; $\eta_{p}^{2}$ = 0.052 | **F _(1,26)_ = 48.412; P < 0.001;** $\boldsymbol{\eta}_{\mathbf{p}}^{\mathbf{2}}$ **= 0.651** | F _(1, 26)_ = 0.645; P = 0.429; $\eta_{p}^{2}$ = 0.024 |

***Table A1. ANOVA analysis results for the factors “training group” and “testing session”.*** *T_max_, maximal plantar flexors torque; VAL, voluntary activation level; EMG_RMS_, plantar flexors activation level; M_max_, maximal M wave; MEP_max_, maximal motor evoked potential; MEP_slope_, slope of MEP input-output curve; MEP_cond_, conditioned motor evoked potential; MEP_test_, non-conditioned motor evoked potential; H_max_, maximal H reflex, H_D1_, conditioned H reflex and H_test_, non-conditioned H reflex. Values in bold denote statistically significant results.*

|  |  |  |  | | |  |  |  | |  |
| --- | --- | --- | --- | --- | --- | --- | --- | --- | --- | --- |
|  |  |  | **Eccentric contraction** | | |  |  | **Isometric contraction** | |  |
|  |  |  | Pre training | | Post training |  |  | Pre training | Post training |  |
|  | **LONgroup** |  | |  |  |  |  |  |  |  |
|  |  | Tmax (N.m) | 114.86 ± 30.80 | | 131.98 ± 33.66 |  |  | 114.82 ± 34.80 | 131.82 ± 36.34 |  |
|  |  | VAL (%) | 81.10 ± 6.05 | | 88.79 ± 3.49 |  |  | 85.83 ± 4.46 | 88.73 ± 3.61 |  |
|  |  | TA Coactivation (%) | 11.46 ± 1.31 | | 11.28 ± 2.03 |  |  | 12.00 ± 1.40 | 11.46 ± 1.31 |  |
|  |  |  |  | |  |  |  |  |  |  |
|  |  | ***SOL*** |  | |  |  |  |  |  |  |
|  |  | ***50% EMG_max_*** |  | |  |  |  |  |  |  |
|  |  | EMG_RMS_/M_max_ | 0.016 ± 0.001 | | 0.023 ± 0.004 |  |  | 0.016 ± 0.002 | 0.022 ± 0.002 |  |
|  |  | MEP_test_/M_max_ | 0.14 ± 0.03 | | 0.14 ± 0.02 |  |  | 0.16 ± 0.01 | 0.14 ± 0.02 |  |
|  |  | M_max (mV)_ | 7.06 ± 1.35 | | 7.76 ± 0.79 |  |  | 7.70 ± 1.89 | 7.74 ± 1.37 |  |
|  |  | M_at_H_max_/M_max_ | 0.16 ± 0.01 | | 0.15 ± 0.03 |  |  | 0.14 ± 0.01 | 0.16 ± 0.02 |  |
|  |  | H_test_/M_max_ | 0.44 ± 0.07 | | 0.41 ± 0.07 |  |  | 0.43 ± 0.05 | 0.42 ± 0.05 |  |
|  |  | ***100% EMG_max_*** |  | |  |  |  |  |  |  |
|  |  | EMG_RMS_/M_max_ | 0.027 ± 0.002 | | 0.038 ± 0.005 |  |  | 0.028 ± 0.004 | 0.039 ± 0.004 |  |
|  |  |  |  | |  |  |  |  |  |  |
|  |  | **GM** |  | |  |  |  |  |  |  |
|  |  | ***50% EMG_max_*** |  | |  |  |  |  |  |  |
|  |  | EMG_RMS_/M_max_ | 0.014 ± 0.002 | | 0.020 ± 0.004 |  |  | 0.016 ± 0.001 | 0.024 ± 0.003 |  |
|  |  | M_max (mV)_ | 7.00 ± 1.09 | | 7.74 ± 0.82 |  |  | 7.88 ± 1.84 | 7.62 ± 1.83 |  |
|  |  | M_at_H_max_/M_max_ | 0.25 ± 0.05 | | 0.27 ± 0.05 |  |  | 0.28 ± 0.06 | 0.27 ± 0.08 |  |
|  |  | ***100% EMG_max_*** |  | |  |  |  |  |  |  |
|  |  | EMG_RMS_/M_max_ | 0.027 ± 0.004 | | 0.039 ± 0.007 |  |  | 0.026 ± 0.006 | 0.039 ± 0.006 |  |
|  | **SHOgroup** |  |  | |  |  |  |  |  |  |
|  |  | Tmax (N.m) | 117.20 ± 27.34 | | 122.48 ± 33.42 |  |  | 112.03 ± 28.4 | 125.65 ± 33.42 |  |
|  |  | VAL (%) | 81.0 ± 5.61 | | 86.67 ± 4.73 |  |  | 88.22 ± 5.07 | 89.43 ± 3.22 |  |
|  |  | TA Coactivation (%) | 11.01 ± 0.73 | | 12.42 ± 1.48 |  |  | 11.73 ± 1.18 | 11.60 ± 1.09 |  |
|  |  | ***SOL*** |  | |  |  |  |  |  |  |
|  |  | ***50% EMG_max_*** |  | |  |  |  |  |  |  |
|  |  | EMG_RMS_/M_max_ | 0.013 ± 0.005 | | 0.024 ± 0.006 |  |  | 0.014 ± 0.003 | 0.021 ± 0.004 |  |
|  |  | MEP_test_/M_max_ | 0.14 ± 0.05 | | 0.15 ± 0.03 |  |  | 0.16 ± 0.02 | 0.14 ± 0.03 |  |
|  |  | M_max (mV)_ | 7.55 ± 2.35 | | 8.23 ± 1.65 |  |  | 7.25 ± 2.05 | 7.76 ±1.43 |  |
|  |  | M_at_H_max_/M_max_ | 0.15 ± 0.09 | | 0.15 ± 0.03 |  |  | 0.14 ± 0.01 | 0.15 ± 0.01 |  |
|  |  | H_test_/M_max_ | 0.44 ± 0.07 | | 0.41 ± 0.07 |  |  | 0.40 ± 0.04 | 0.43 ± 0.02 |  |
|  |  | ***100% EMG_max_*** |  | |  |  |  |  |  |  |
|  |  | EMG_RMS_/M_max_ | 0.027 ± 0.001 | | 0.038 ± 0.005 |  |  | 0.027 ± 0.004 | 0.037 ± 0.005 |  |
|  |  |  |  | |  |  |  |  |  |  |
|  |  | ***GM*** |  | |  |  |  |  |  |  |
|  |  | ***50% EMG_max_*** |  | |  |  |  |  |  |  |
|  |  | EMG_RMS_/M_max_ | 0.014 ± 0.002 | | 0.022 ± 0.004 |  |  | 0.015 ± 0.002 | 0.023 ± 0.004 |  |
|  |  | M_max (mV)_ | 7.76 ± 2.40 | | 8.41 ± 1.65 |  |  | 7.36 ± 2.04 | 8.16 ± 1.60 |  |
|  |  | M_at_H_max_/M_max_ | 0.29 ± 0.09 | | 0.29 ± 0.05 |  |  | 0.28 ± 0.03 | 0.29 ± 0.09 |  |
|  |  | ***100% EMG_max_*** |  | |  |  |  |  |  |  |
|  |  | EMG_RMS_/M_max_ | 0.029 ± 0.006 | | 0.039 ± 0.004 |  |  | 0.028 ± 0.005 | 0.038 ± 0.007 |  |
|  |  |  |  | |  |  |  |  |  |  |

***Table A2. Effect of eccentric training at two different muscle lengths on maximum performance and electrophysiological properties.*** *T_max_, VAL, EMG_RMS_/M_max_*, *Coactivation, MEP_test_/M_max_, M_max_ (MEP), M_max_ (H), M_at_H_max_/M_max_, and H_test_/M_max_ ratios of SOL and EMG_RMS_/M_max_, M_max_ (MEP), M_max_ (H), M_at_H_max_/M_max_ ratio of GM, according to training group, contraction type and testing session.* *T_max_, maximal plantar flexors torque; VAL, voluntary activation level; EMG_RMS_, plantar flexors activation level; M_max_, maximal M wave amplitude; MEP_max_, maximal motor evoked potential; H_max_, maximal H reflex and H_test_, non-conditioned H reflex.*
